# Engagement of Nepalese High-School Students in Cancer Awareness Using a Photovoice Based on the Health Belief Model

**DOI:** 10.3390/healthcare9101283

**Published:** 2021-09-28

**Authors:** Kritika Poudel, Naomi Sumi

**Affiliations:** 1 Center for Environmental and Health Sciences, Hokkaido University, North 12, West 7, Sapporo 060-0812, Japan; kpoudel@hs.hokudai.ac.jp; 2 Faculty of Health Sciences, Hokkaido University, North 12, West 5, Sapporo 060-0812, Japan

**Keywords:** cancer perception, high school students, adolescents, photovoice method, Nepal

## Abstract

Delivering cancer education is one of the strategies for implementing health promotion and disease prevention programs. Moreover, cancer education can help people understand the risks related to specific behaviors that can trigger cancer during later life stages. This study examines the cancer perception of high school students (median age: 14 years) using a photovoice based on the health belief model. Students were requested to take photographs to answer the framework question: “How is cancer present in your surrounding?” A theoretical thematic analysis was conducted to develop codes, and the narratives completed by the participants on the SHOWED checklist were used to create contextualization. With the use of the health belief model, the following factors were determined: risk factors and environmental pollution (perceived susceptibility), symptoms (perceived severity), prevention and screening (benefits), ignorance and poverty, and ineffective laws and regulations (perceived barriers). Linked to these themes, students’ narration demonstrated the risk of developing cancer if the same environment and inadequate regulations persisted. This study highlights the need to incorporate the participation of adolescents in the design, implementation, and monitoring of several community issues to help address several unanswered questions.

## 1. Introduction

Adolescent health is described as a different range of approaches for the prevention, detection, or treatment associated with young people’s health and well-being [1]. The comprehensive adolescent health package includes promoting mental health and wellbeing, a safe environment, improving access to nutritious and healthy food, mitigating the negative consequences associated with substance abuse, promoting physical activity, and improving healthcare-seeking behaviours in communities. A multidimensional approach is required to safeguard adolescent health, such as through families to protect and nurture, schools to promote healthy development, healthcare that is responsive to the needs of adolescents, a safe transport system, and laws to protect adolescents’ rights [2].

Approximately 23.6% of Nepal’s population comprises adolescents aged 10–19 years [3]. During adolescence, young people acquire new habits and behaviors. Various risk factors, such as alcohol and tobacco use, inadequate physical activity, unprotected sexual habits, or violence can threaten their current and future health as they reach adulthood [4]. Although most instances of adolescent morbidity and mortality are preventable, adolescents face several barriers in accessing health information and services [5]. Furthermore, there is an alarming issue of sexual and reproductive health concerns among adolescents in Nepal [6]. Although many studies have been conducted to understand issues related to sexual and reproductive health, substance abuse, and school-based health programs [7,8], there is limited evidence to address cancer awareness in Nepal. Increasing cancer awareness among adolescents can supplement their knowledge and aid in recognizing cancer symptoms early and search for timely medical help during adolescence and adulthood [9]. Providing health education is one of the strategies for implementing health promotion and disease prevention programs. Cancer education can help adolescents understand the risks related to specific behaviors that can trigger the origin of cancer during later life stages. Repetitive and streamlined education programs focused on diseases may further help students understand the association between specific habits and cancer. However, these habits do not persist for an extended period, thereby rendering this method ineffective [10,11]. Furthermore, cancer education programs have been proven to be effective when adolescents, peer groups, and families easily share opinions [9,10,12,13,14].

The health belief model (HBM) was developed during the early 1950s and remains one of the popular models used in health education programs. HBM includes four major subscales—perceived susceptibility, severity, benefits, and barriers. Several factors determine the likelihood that an individual will act to prevent or detect disease: perceived susceptibility to the health condition, perceived severity of the health threat, perceived benefits of performing the health behavior, and perceived barriers to performing this behavior [15,16]. In addition, there are two other subscales: cue to action and self-efficacy. In this study, we used the four major subscales to understand the cancer perceptions of participants through a photovoice.

A photovoice is the process of used to identify, represent, and enhance a community through photographic methods. Previously known as photo novella, the photovoice approach has been used for various community-based participatory research (CBPR) projects, primarily to enable people to record and reflect on different aspects of the community, promote critical knowledge through discussions of photographs taken, and ensure that their concerns are addressed by policymakers [17]. Photovoices allow participants to actively engage with researchers in the research process and enable the exchange of opinions and experiences [18]. Various studies have used photovoices as a research methodology to explore the thoughts of participants and empower local assessment [19,20]. Additionally, photovoices are used as a pedagogical tool to examine the learning process of the students as well as to introduce qualitative research methods to community members. These studies helped to record the voices of individuals and their visions for their lives and communities.

Although some studies have used a photovoice as a teaching tool in different classrooms [21,22,23], to date, a photovoice has not been utilized in cancer education research. The photovoice method encourages participants to engage in the research process and provides access to subjective experiences that are difficult to address from the outside; thus, we aimed to understand student perceptions regarding cancer by using a photovoice among school students.

## 2. Materials and Methods

This qualitative study was conducted in different schools in Lalitpur metropolitan city, Nepal. Convenience sampling was used to select schools for data collection. Permission to conduct this study was obtained from the school principal, teachers, parents, and students. High school students in the same grade were recruited as study participants.

### 2.1. Photovoice Research Method

Different exercises were conducted to ensure students were familiar with the photovoice.

1. Training: The researchers introduced the aim and the photovoice process to the participants. A 90-min training session was provided regarding the photovoice and its purpose in the current study, camera usage, and photography ethics. Participants were requested to obtain informed consent when taking photographs of people.
2. Data collection: The SHOWED checklist was provided to participants. The researchers explained to participants how they could present their opinions about the photos based on the SHOWED checklist [24].

The questions in the SHOWED checklist are as follows:

1. What do you see here?
2. What is really happening here?
3. How does this relate to our lives?
4. Why does this problem or this strength exist?
5. What can we do about this?

Participants were divided into a group of six students. The framing question “How is cancer present in your surrounding?” was posed to them. Each group was given three days to take photos and answer the questions. They were requested to select photos that they perceived as the most meaningful as well as those that represented their thoughts. 

### 2.2. Group Discussion by Theme

Each student discussed their best representative photos with their respective groups. Following this, the researcher facilitated a comprehensive discussion. Students were requested to relate the narration of each photo to their groups. Based on the discussion, groups were requested to develop a poster. Each group was allocated five minutes to describe their posters in front of all the groups. 

#### Data Analysis

After the photovoice session, a theoretical thematic analysis was conducted to develop the codes. The narratives completed by the participants on the SHOWED checklist [17,25] were used to develop contextualization. The codes were generated and modified to create the themes. These themes had to directly address the framing question: “How is cancer present in your surrounding?”. Rather than the photographs, the narration was analyzed to develop themes. The researchers maintained neutrality to ensure the situations were not intentionally influenced or manipulated.

## 3. Results

### 3.1. General Characteristics of Participants

**Figure 1:**
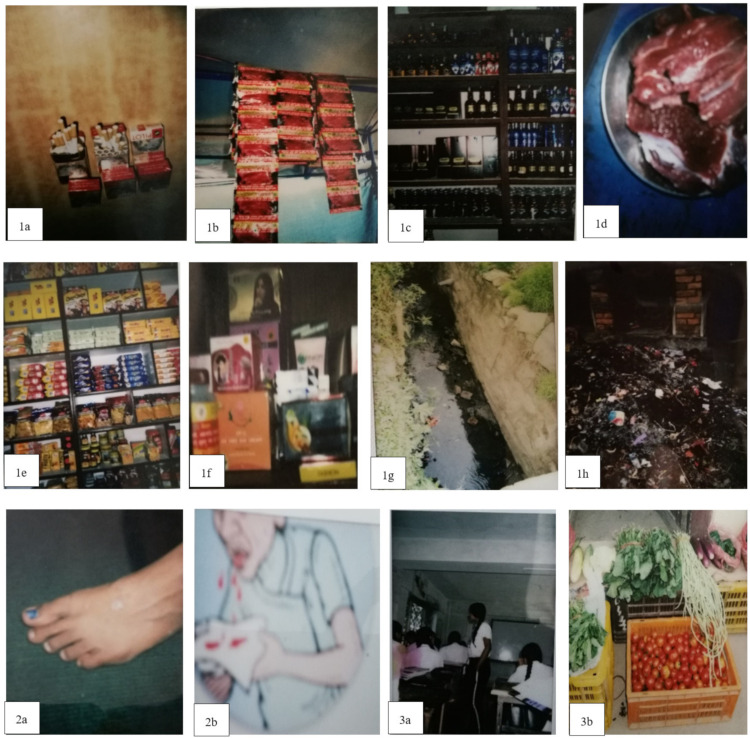

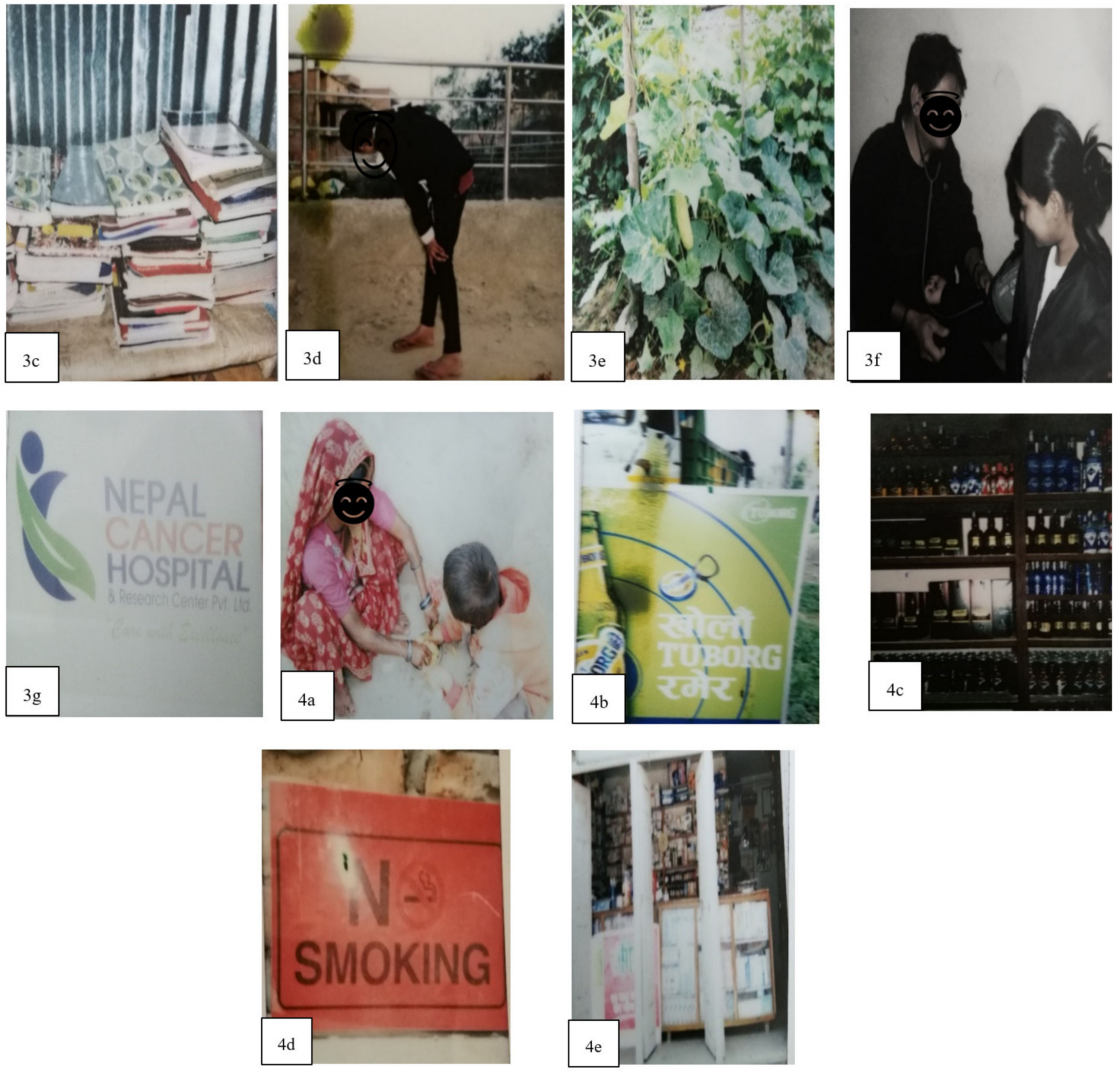
Photovoice photographs (numbered by health belief subscales).

A total of 152 students participated in this study through convenience sampling. The median age of the participants was 14 years, and approximately 53.9% (N = 82) were female. A total of 30.3% (N = 46) of participants had talked about or discussed cancer with their friends/families, and around 79.6% (N = 121) of participants had considered cancer talk as an important factor in cancer education. Figure 1 presents the photographs selected by the participants. The narratives are presented in Table 1. Based on the narration, the photographs were divided into different themes based on the HBM. Although not directly associated, participants also emphasized environmental factors, such as air pollution, sewage, and improper waste management as risk factors for various diseases.

### 3.2. Defining Cancer Perception: How Is Cancer Present in Your Surrounding?

#### 3.2.1. Cancer and Susceptibility

Participants described different risk factors that can increase the susceptibility of cancer risk, thereby creating the sub-theme “Risk factors” (see Figure 1 [1a–f]; Table 1 [1a–f]) and environmental pollution (see Figure 1 [1g–h]; Table 1 [1g–h]). Participants discussed different risk factors such as tobacco (smoking and chewing), alcohol consumption, excessive consumption of red meat, high intake of processed and junk foods, and use of different cosmetics that can increase the susceptibility of cancer. Students also highlighted garbage and open sewage pipes, which can increase the risk of waterborne diseases in their communities. Although Figure 1 [1g–h] is not related to cancer susceptibility, students assumed that these environmental pollutants could contaminate water, soil, and air, thereby leading to unhygienic living areas that could trigger various diseases.

#### 3.2.2. Cancer and Severity

Participants underlined different potential symptoms of cancer, thus creating the sub-theme “Symptoms” (see Figure 1 [2a–b]; Table 1 [2a–b]). Changes in skin color, size of warts and moles, and blood noted while coughing were discussed. During the narration, participants focused on the severity of cancer and the importance of properly monitoring symptoms during the early diagnosis of cancer.

#### 3.2.3. Cancer and Benefits

Two sub-themes, “Prevention” and “Screening,” were developed based on the narration of participants. The sub-theme “Prevention” (see Figure 1 [3a–e]; Table 1 [3a–e]) highlighted the importance of cancer education, healthy diet, exercise, reading books, and consumption of organic foods. Another sub-theme, “Screening,” focused on regular check-ups and hospital access for treatments. Participants were aware of the different services being provided by the hospitals and their role in screening for diseases.

#### 3.2.4. Cancer and Barriers

Participants emphasized different aspects of their community that can serve as a barrier to cancer awareness and treatment. Two sub-themes were developed based on the narration: “Ignorance and Poverty” and “Ineffective law and regulations.” Under the sub-theme “Ignorance and Poverty” (see Figure 1 [4a]; Table 1 [4a]), participants noted the medical and treatment services being expensive, such that all citizens could not access them. They also questioned whether cancer screening tests are conducted free-of-charge. The sub-theme “Ineffective laws and regulations” (see Figure 1 [4b–e]; Table 1 [4b–e]) highlighted the ineffective laws related to buying, selling and hoarding alcohol, as well as the availability of medicines without prescription. Participants criticized the actions of the government with regards to regulating advertisements for tobacco and alcohol on social media.

### 3.3. Cancer Perception and Health Belief Model

**Figure 2:**
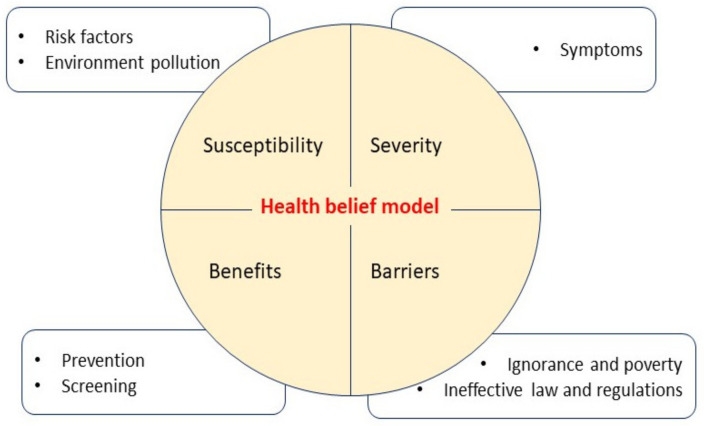
Defining the cancer perception via health belief model.

Figure 2 presents a summary of the thematic analysis through the HBM. Participants’ narratives and codes linked the four subscales of the HBM. Figure 2 was developed based on the themes derived from the SHOWED checklist responses of participants. Participants were susceptible to various risk factors that can contribute to developing cancer. They perceived symptoms as the severity of the diseases and perceived prevention and screening as the benefits of cancer treatment. Ineffective law and regulations and poverty were considered barriers to cancer prevention. (INSERT FIG 2).

## 4. Discussion

The purpose of this study was to understand the perceptions of high school students regarding cancer by using the photovoice process. We examined how adolescent students perceived cancer in their community in terms of the risk factors, preventive measures, or health barriers. This study also included cancer communication among friends and discussions regarding cancer while getting involved in the photovoice process. These students were adolescents and thus understood the importance of peer influence among those in this age group; therefore, we formed groups to conduct the photovoice. Several studies have revealed the role of peers in directly and indirectly shaping health behaviors, well-being, and perceptions about school [26]. A study showed that peers play an important role in influencing risk or protective behaviors [27]. Our previous study also supported the idea of peer group discussion in encouraging active learning and problem-solving through more sustainable methods [10]. 

In the current world, adolescents are actively engaged in different types of digital media. Social media can provide an opportunity for adolescent education, self-expression, creativity, and entertainment [2]. Photovoices have been used as a research tool among adolescents in various studies. Studies have used photovoices to promote social action and civic participation among adolescents to improve school conditions [28,29]. In this study, we used a photovoice as a tool to understand the opinions of students regarding cancer. Participants discussed various risk factors, symptoms, and preventive measures to address cancer. Studies have recommended reducing tobacco use, increasing physical activity, controlling weight, improving diet, limiting alcohol consumption, conducting routine cancer screening tests, and avoiding excessive exposure to the sun to reduce cancer risks [30]. Unless perceived susceptibility is seriously considered, risky health-related behaviors may endure. Students in this study perceived several factors that increased the susceptibility of developing cancer. Therefore, if specific cues to action are introduced, adolescents have the potential to initiate healthy habits in society. These cues could be communicated through a message on a poster, calendar, playing cards, or a message reminder on any social media [31].

The government in Nepal has attempted to increase access to health care services to achieve high coverage. This study highlighted expensive medical services, ignorance and poverty, and a lack of strict rules and regulations as barriers to healthcare services. These findings are supported by another Nepali study that focused on inequality, poverty, traditional and cultural practices, and the heavy burden placed on healthcare professionals as the primary reason for poor health service quality [32]. Another Nepali study emphasized the unavailability of medicine at the health post hospitals while seeking healthcare services [33]. Barriers to health services and healthcare delivery require urgent intervention. Strengthening the health system and providing equitable distribution of health services must be endured by improving the efficiency of the hospital, monitoring unaffordable payments, motivating health workers, and building beneficial packages to cover major health services, including non-communicable diseases [34].

The study participants explored their understanding of cancer in their surroundings. The findings from the photovoice are related to practice, research, and education. It helped the participants identify health priorities and existing social problems around them. Decision-making on expenditure to healthcare heavily depends on the individual’s health status and the certainty about the future [35]. It is more challenging to people in the low-income countries where people have to pay high cost from their pockets for the basic treatment without any health insurance support. Therefore, photovoices can be more effective in the context of low-resource setting where early detection of health problems can help to lower the burden of diseases on people [36]. Photovoices as a learning process can be considered a valuable tool for behavioural change interventions, focusing on identifying problems in society and providing a platform for unheard voices. Therefore, linking behavioural change techniques with CBPR can help to sustain behavioral change at the community level [20,37].

Various studies have suggested the need for schools to understand school community participation, its importance, and execution [38,39]. School-based health promotion interventions can include the photovoice method for health education and can address various public health-related issues in school and community-based settings. Application of the photovoice method within a school setting can help schools achieve healthy promoting environments more efficiently [40]. Within the classroom setting, teachers can flip the role of expert to being a facilitator and stimulate critical thinking of students and empower student’s participation. Schools and communities must work together to foster capacity-building among students regarding their communities.

Visual methods are increasingly used in different disciplines. Visual methodologies are used to understand and interpret images, which includes photography, film, video, drawing, sculpture, artwork, graffiti, and cartoons. These methodologies are a new approach to qualitative research that have added another dimension to pre-existing methods by creating knowledge, which is becoming popular and beneficial in health and illness research [41]. Pictures closely linked to written text can effectively increase attention to and recall health education information more effectively than text alone [42]. In particular, people with low literacy skills are likely to benefit, and it is even easier to attract children and adolescents who are more likely to choose pictures over text. However, the promise of a photovoice as a robust visual research methodology and pedagogy lies in the rigor, trustworthiness, and adaptation of the project design and process [43].

Adolescents have a sense of self-worth and self-esteem, and thus have the confidence to express their views and act as change agents in the community [44]. The researcher played a process-facilitating role while participants engaged in the research process. Through this study, we can understand the perceptions and knowledge of adolescents regarding their surroundings, communication with each other, critical thinking, and problem-solving skills. This study suggests that adolescents should be involved in the planning, monitoring, and evaluation of health services and in decisions regarding their care. Moreover, photovoices can serve as a method for achieving results by empowering adolescents themselves to be change agents. 

This study has several strength and limitations. This study involved students in the research process, which provided an opportunity to understand their concerns regarding cancer in their surroundings. The sharing of ideas and discussions among students increased their engagement in critical thinking, which helped them to achieve a deeper understanding and create a sense of responsibility toward their communities. However, the photovoice method requires time, effort, and commitment from the study participants. In addition, training sessions and group meetings must be conducted prior to actions to discuss and broaden the concept of learning. The researcher serves as a facilitator by supporting and guiding the data collection process; therefore, the findings may sometimes deviate from what the researchers intended to measure.

## 5. Conclusions

This study supports the adoption of a photovoice as a method for identifying cancer perception among high school students. The photovoice provided an opportunity for students to engage in the research process and explore their surroundings and discuss their perceptions of cancer in their areas. Students identified several barriers to health services, including gaps in laws and regulations. A deeper understanding of the learning process created a genuine and sustainable learning experience for participants. Incorporating a photovoice in the design, implementation, and monitoring can help address several unanswered questions. In addition, using a photovoice in collaboration with family members, community-based clubs, and local organizations can increase community engagement and allows for more empowered and sustainable actions. Further extensive studies are crucial for evaluating the long-term impact of photovoice on individual or community health.

## Figures and Tables

**Table 1:** Selected summary of photovoice narratives by participants (please refer to Figure 1).

Photo		What Do You See Here?	What Is Really Happening Here?	How Does It Relate to Our Lives?	Why Does This Situation Exist?	What Can We Do about It?
1	a	I see many cigarette packets.	People buy and smoke these cigarettes.	It is related to our body because it can damage our lungs and cause cancer.	There are many reasons. Some people smoke to show off and some do it to reduce stress. In the past, many people used to smoke tobacco, so this generation also copied this behavior from their family members and started smoking. Cigarette smoke can affect other people nearby, even us.	People should stop smoking. We need to ban cigarette production and distribution in our community.
	b	Chewing tobacco is sold.	They are arranged attractively for sale in the shop.	Eating and chewing tobacco can cause mouth and oropharynx cancer.	Sometimes people tend to chew tobacco due to stress or to relieve tension. Although there are warnings on the packet that it is injurious to health, people still buy and chew them.	It is very important to increase awareness about healthy and unhealthy foods. We should ensure people are aware about the effects of chewing tobacco.
	c	Bottles of different alcohol in the shop.	The sale of alcohol has continued.	If we consume large amounts of alcohol, it can damage our liver and increase the risk of contracting diseases.	There is a huge demand for alcohol in our society. To fulfill this demand, the shopkeeper provides more alcohol for sale. Although this is his business, it is ruining the lives of others.	We should encourage people to limit their alcohol intake. The government should levy hefty tax on these shops.
	d	Red meat is openly sold.	High and frequent consumption of red meat can harm our body.	Consuming red meat daily in large amounts is not a healthy habit.	Red meat, such as pork and buffalo meat, have been consumed since the ancient days due to the lack of items available for consumption. Recently, people began eating grilled or burnt meat which can cause indigestion and stomach diseases and increase the risk of cancer.	Only a limited amount of red meat should be consumed. We need to focus on having a healthy and balanced diet. We need green leafy vegetables and fruits for vitamins and minerals.
	e	I see attractive packaged and processed foods at the shop.	These packets contain unhealthy, junk foods that are not good for one’s health.	The packages are eye-catching, so people are drawn to them. These products contain processed chemicals that are harmful to our body.	The junk foods have attractive packaging and are advertised on television and radio. These advertisements are very attractive to both kids and adults, so it encourages people to buy and eat those unhealthy foods.	We need to limit junk food intake. These foods contain a large amount of salt and chemicals and increase the chance of obesity. The sale and intake of these food items should be limited. We need to focus on healthy diets.
	f	Different facial creams and unnecessary soaps.	Someone bought it to use it on their skin.	Using facial creams and unnecessary soaps on our skin can lead to various skin problems. Many skin infections may lead to cancer.	This situation exists because of advertisement on television, radio, and so on. People are encouraged to use cosmetic products to attain beautiful skin. Excessive use of low-quality cosmetics can increase the risk of skin cancer.	We should make people aware they should not use unnecessary facial creams. Provide them with information about using ayurvedic soaps and creams. If we use unnecessary soaps and creams, it can lead to skin diseases.
	g	I can see filthy garbage and sewage leaking from pipes and ruining the land nearby.	Waste and sewage are leaking from pipes and producing a foul smell. It causes land pollution, and foul smells.	This is creating a significant problem among local people. It is harming living beings and causing various types of pollution. People are suffering from water borne diseases due to pollution.	A large number of people are living in this area; thus these drainage pipes help to drain sewage and other liquids. However, they are not properly managed. Whenever they leak, it causes serious health problems and foul smells in the surrounding area.	Sewage and waste management should be conducted in a timely manner. If these pipes leak, we are the ones who suffer, so we need to observe and check the condition of large drainage pipes in our society. Once it leaks or starts having small problems, we need to fix it immediately.
	h	Garbage is being dumped openly.	Waste and garbage is dumped in open areas.	Improper waste disposal leads to air, noise, and soil pollution.	Improper waste disposal has created pollution in various areas. There is a foul smell everywhere. Several worms and flies are visible on the streets. People are suffering considerably due to air, soil, and noise pollution. It is increasing the risk of various communicable diseases such as diarrhea, cholera, etc.	Waste should be properly dumped. Sewage should not leak to the local areas. Everyone should work on reducing pollution. It is our responsibility.
2	a	Patches and moles can be present in our body.	Moles, patches, and warts should be monitored for change in color and size.	Increase in size and change in color of the mole, warts can be warning signs of cancer.	We need to check our body and regularly monitor them. Our parents or family members can have some irregular moles or warts, patches which should be monitored.	Timely monitoring of symptoms is important.
	b	I see poster of symptoms related to cancer.	The flyer contains information about different symptoms of cancer.	The flyer is trying to create awareness among public about the cancer.	People are ignorant about the signs and symptoms. They tend not to care about their body. They go to the hospital when they almost reach the last stage.	Properly monitoring signs and symptoms can help to ensure easy and early diagnosis.
3	a	Cancer education is being provided.	Students are receiving information about cancer.	Cancer education helps students to understand cancer.	Schools and colleges are encouraging students to understand the risk factors, symptoms, and measures for the prevention of cancer.	It is important for schools to teach students about cancer and its preventive measures.
	b	Healthy diet is important for health	Green vegetables and fruits are being sold.	It is very important to consume green leafy vegetables and fruits.	Green leafy vegetables are important sources of vitamins and minerals.	We should encourage people to consume green leafy vegetables and fruits.
	c	A girl is exercising.	A young girl is doing some type of exercise.	Exercise plays a vital role in keeping our body fit. It helps us to prevent diseases.	Exercise is an important part of human life. Recently, people are becoming sedentary and do not have time to exercise. However, this will only make us unhealthy and fat.	We must perform different types of exercise for at least 15 min per day. Life is short and we should not ruin it by not staying fit.
	d	I see books.	Books are the source of education.	We go to school to learn new things every day. Books provide considerable information about diseases and health.	We need to develop the habit of studying and reading more books. Newspapers and posters ae also another source of information. They provide the latest information about diseases, drugs, discovery, and challenges.	Everyone needs to know about and read books. We can teach our family by sharing information from books. Books are an important part of a student’s life.
	e	I see green plants and cucumber.	People are growing organic foods using fewer pesticides.	People presently use pesticides and chemicals to improve food quality. In reality, they are only ruining the originality of these foods.	The use of pesticides may increase the attractiveness of foods but decrease the food quality. We should not consider the outer beauty of food while buying it but should consider our health instead. Organic foods are healthy, and we should grow or buy them. Pesticides and chemicals harm our body directly and indirectly.	If we stop buying foods grown using pesticides, farmers will reduce the use of chemicals to grow foods. Then we will get healthy foods and improve our health condition.
	f	I see someone is checking their blood pressure at the health post nearby.	A person is checking their blood pressure and is eager to know their blood pressure level.	We must have a normal blood pressure range. High or low blood pressure is risky to health.	This person wants to know their blood pressure; thus, he is at a regular check-up. People should have regular checkups to monitor their weight, blood pressure, and sugar level.	This is preventive measures. We should check our blood pressure timely. Also, we should maintain our salt intake. Regular checkup helps to prevent diseases.
	g	I see hospital posters.	We should go to the hospital without delay.	Many people do not go for treatment in the early stage even when hospitals are nearby.	Hospitals are there for people. For timely diagnosis and specialized treatment, people need to go to hospital early. They should not depend on neighbors talks and traditional beliefs.	Many people seek advice from family, neighbors and follow their advice even if they are seriously ill. We must educate people about going to hospital on time and not delaying their treatment. Health is wealth.
4	a	A mother feeding her child.	A mother is buying street foods and feeding her child.	Junk foods are cheaper and other foods are expensive. Ignorant, illiterate, and poor people do not have money to eat healthy foods.	Along with illiteracy, ignorance and poverty, is a big problem in Nepal. Many poor people do not have money to buy good and healthy foods. They also do not know importance of eating healthy foods, so they eat low quality food and cheaper foods. This affects their health, but they do not have the money to eat healthier foods. Even when they fall sick, they do not have access to health services.	Cancer awareness programs should be conducted. The government should reduce the prices of vegetables, education, and medical treatment so that everyone can access to them. Is the screening test for cancer free?
	b	There is an advertisement of beer in the photo.	Some beer companies are advertising their beer.	It influences many people to buy and consume beer. Alcohol affects our health badly.	Advertisements attract young and old people to buy beer. These advertisements are everywhere in the market. Many students hid and drink alcohol because their advertisements are attractive.	Why is the government showing these advertisements on the television, radio, boards? Can we ask the government to ban the sale of alcohol?
	c	The shop has variety of alcohol.	Everyone can go and buy alcohol.	We can buy alcohol from the shop if we say it is an order from our parents.	There are no strict laws or rules for buying alcohol. Anyone can go and buy it. Although we cannot drink alcohol because we are under 18 years of age, we can hide and do whatever we want.	Shopkeepers should not allow children to buy alcohol, even if it is for their parents’ consumption. They also should not allow children to buy cigarettes for their parents.
	d	I see a non-smoking poster.	The government has put non-smoking posters in some areas.	This poster discourages people from smoking in these areas.	Although these posters are there, people do not care and still smoke in front of the poster.	Giving punishments and penalties to those who do not follow the rule.
	e	I see a medical pharmacy.	Lots of medicines are available for sale.	After treatment, many people need to take medicines, but they are expensive.	When people are unhealthy, they do not go to hospital on time, thus, delaying treatment. Also, the medicines and treatments for cancer are expensive. Many people cannot afford these medicines, so they do not go to the hospital unless they are very sick. Also, people can buy many medicines directly without a doctor’s prescription.	The government should decrease the price of expensive medicines. The pharmacist should not give medicines to people without a prescription.

## Data Availability

The data are not publicly available due to ethical restrictions and specific legal framework in Japan. All inquiries should be addressed to Naomi Sumi, corresponding author of this paper.
